# Six genes involved in prognosis of hepatocellular carcinoma identified by Cox hazard regression

**DOI:** 10.1186/s12859-021-04095-7

**Published:** 2021-03-30

**Authors:** Qinghong Dai, Tao Liu, Yongchao Gao, Honghao Zhou, Xiong Li, Wei Zhang

**Affiliations:** 1 Shenzhen Center for Chronic Disease Control, Shenzhen, People’s Republic of China; 2 The First Affiliated Hospital of Guangdong Pharmaceutical University, Guangzhou, 510060 People’s Republic of China; 3 Department of Clinical Pharmacology, Xiangya Hospital, Central South University, 87 Xiangya Road, Changsha, 410008 People’s Republic of China; 4 Institute of Clinical Pharmacology, Central South University, Hunan Key Laboratory of Pharmacogenetics, 110 Xiangya Road, Changsha, 410078 People’s Republic of China; 5 Engineering Research Center of Applied Technology of Pharmacogenomics, Ministry of Education, 110 Xiangya Road, Changsha, 410078 People’s Republic of China; 6 National Clinical Research Center for Geriatric Disorders, 87 Xiangya Road, Changsha, 410008 People’s Republic of China

**Keywords:** Cox hazard regression, DEGs, HCC, Hub gene, Risk score, Prognostic model

## Abstract

**Background:**

Hepatocellular carcinoma (HCC), derived from hepatocytes, is the main histological subtype of primary liver cancer and poses a serious threat to human health due to *the high incidence* and *poor prognosis*. This study aimed to establish a multigene prognostic model to predict the prognosis of patients with HCC.

**Results:**

Gene expression datasets (GSE121248, GSE40873, GSE62232) were used to identify differentially expressed genes (DEGs) between tumor and adjacent or normal tissues, and then hub genes were screened by protein–protein interaction (PPI) network and Cytoscape software. Seventeen genes among hub genes were significantly associated with prognosis and used to construct a prognostic model through COX hazard regression analysis. The predictive performance of this model was evaluated with TCGA data and was further validated with independent dataset GSE14520. Six genes (*CDKN3, ZWINT, KIF20A, NUSAP1, HMMR, DLGAP5*) were involved in the prognostic model, which separated HCC patients from TCGA dataset into high- and low-risk groups. Kaplan–Meier (KM) survival analysis and risk score analysis demonstrated that low-risk group represented a survival advantage. Univariate and multivariate regression analysis showed risk score could be an independent prognostic factor. The receiver operating characteristic (ROC) curve showed there was a better predictive power of the risk score than that of other clinical indicators. At last, the results from GSE14520 demonstrated the reliability of this prognostic model in some extent.

**Conclusion:**

This prognostic model represented significance for prognosis of HCC, and the risk score according to this model may be a better prognostic factor than other traditional clinical indicators.

## Background

Liver cancer represent currently the sixth most frequent malignancy and the second mortality of cancer-related deaths, with more than 85,000 new cases annually in the world. HCC accounts for approximately 85–90% of liver cancer(1). The majority of HCCs occur in patients with underlying chronic liver disease and the main risk factors are the presence of hepatitis virus, alcohol abuse, obesity, *nonalcoholic* steatohepatitis and metabolic syndrome(2). Currently available treatments for HCC include *surgical resection*, *liver transplantation, chemotherapy,* radiofrequency ablation and the multikinase inhibitor sorafenib(3). However, only a small part of patients are eligible for these therapies, and the clinical efficacy is also variable and very limited for advanced HCC due to the inherent biological and genetic heterogeneity(4). Given the high incidence and mortality of HCC, which lead to serious *health problems and heavy social burden*, identifying new biomarkers to further reveal pathogenesis, predict clinical prognosis and provide individualized treatment for HCC patient are critical and urgently demanded.

The rapid development of high-throughput technology make the researches of disease-related biomarker more and more feasible and reliable(5). Generally, the occurrence and further development of tumors are caused by multiple gene abnormalities, so it is difficult for a single gene to accurately reflect the tumor characteristics. Recently, there was a view that using multiple genes to predict tumor biological features seems more convincing(6, 7). The purpose of this study was to use the gene expression data in Gene Expression Omnibus (GEO) and The Cancer Genome Atlas (TCGA) database to develop a multigene model to predict the prognosis of patients with HCC.

In this study, three GEO datasets were used to screen out hub genes. Then, a prognostic model was constructed using TCGA data on the basis of these hub genes and the predictive performance of this model was evaluated. Finally, an independent GEO dataset was further used to validate the significance of this model. All processes of this study were based on R, Perl software and several online tools.

## Methods

### Dataset preparation

In this study, three raw gene expression profiles (GSE121248, GSE40873, GSE62232) were downloaded from GEO database (https://www.ncbi.nlm.nih.gov/geo) (8). GPL570 (HG-U1331_Plus_2) Affymetrix Human Genome U133 Plus 2.0 Array was performed for these datasets. The fragments per million (FPKM) expression profile of 424 HCC samples were retrieved from TCGA database (https://cancergenome.nih.gov/). In addition, GSE14520 was used as validation cohort. Table 1 listed the sample size of each dataset.

**Table 1:** Sample size of each dataset

Data set	Non-tumor samples	Tumor samples
GSE121248	37	70
GSE40873	49	0
GSE62232	10	81
GSE14520	241	247
TCGA	50	374

### Data preprocessing and identification of DEGs

The raw data of gene expression profiles from GEO were preprocessed for background correction, log2 transformation, quantile normalization and then probeset summarization to gain gene expression matrix by using the Robust Multi-array Average (RMA) algorithm of the “affy” R package(9). GSE62232 and GSE40873 were merged into an merged dataset by Perl due to the scant nontumor samples in GSE62232, and no tumor samples in GSE40873. Given the batch effects in two datasets, the ComBat algorithm of the “sva” R package was employed to remove batch effects(10). The DEGs of the merged dataset and GSE121248 were analyzed through the Empirical Bayes function in “limma” R package(11), with the thresholds of adjust p < 0.05 and log fold changes (log FC) > 2.0. Visualization of the overlapping genes among the DEGs of the merged dataset and GSE121248 was achieved by online software VENNY (https://bioinfogp.cnb.csic.es/tools/venny/).

### Construction of PPI network and identification of hub genes

The Search Tool for the Retrieval of Interacting Genes database (STRING, http://string-db.org) was utilized to construct PPI network with interaction score >  = 0.7 based on the DEGs(12). The subnetworks were generated by Molecular Complex Detetion (MCODE) with *default* parameters, a plugin for Cytoscape software used for clustering a significant subnetwork in the PPI network to screen hub genes(13).

### Differential expression and functional enrichment of hub genes in TCGA cohort

HCC samples in TCGA cohort were uesd to perform differential expression analysis, Gene Ontology (GO) enrichment analysis (achieved by “enrichiplot” and “org.Hs.eg.db” R packages) and Kyoto Encyclopedia of Genes and Genomes (KEGG) pathway analysis (achieved by “digest” and “Goplot” R packages), which aimed to explore the possible functions of the hub genes. Functional categories with FDR < 0.05 and log FC > 2.0 were considered as significant pathways.

### Construction of the prognostic model and predictive performance evaluation

**Fig. 1:**
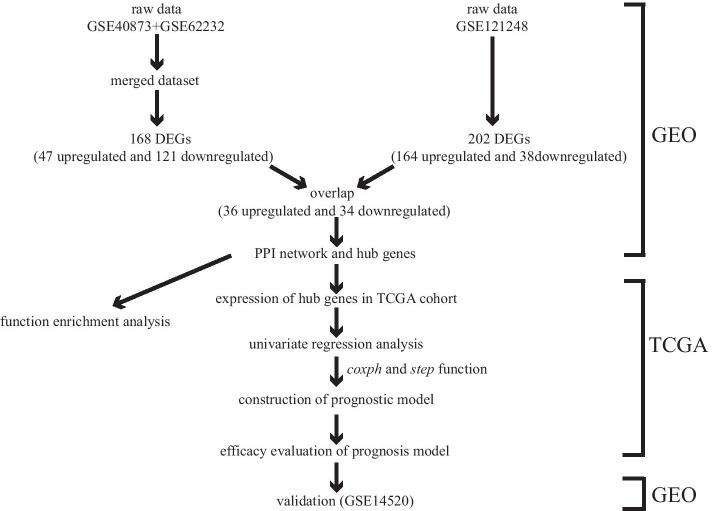
Overall process of this study. MCODE, a plugin for Cytoscape

Hub genes that could predict prognosis independently (P < 0.05) in univariate hazard regression analysis, were used to construct the prognostic model through COX hazard regression. The initial construction of the model employed *coxph* function of the “survival” R package, and the subsequent optimization of the model used the *step* function. Genes with P < 0.1 were included in the model, and risk score was equal to the sum of the product of the expression value of each gene and its correponding hazard coefficient. The risk scores of TCGA samples were calculated and these samples were divided into high- and low-risk groups according to the median of risk score for subsequent evaluation of the model performance. KM survival curve, risk score analysis, independent prognostic analysis and ROC curve were implemented to evaluate the performance of this model, and the correlation between risk score and survival state was also analyzed. At last, the predictive value of the model was validated by GSE14520. The overall workflow of this study was shown in Fig. 1.

## Results

### Identification of DEGs and hub genes

**Fig. 2:**
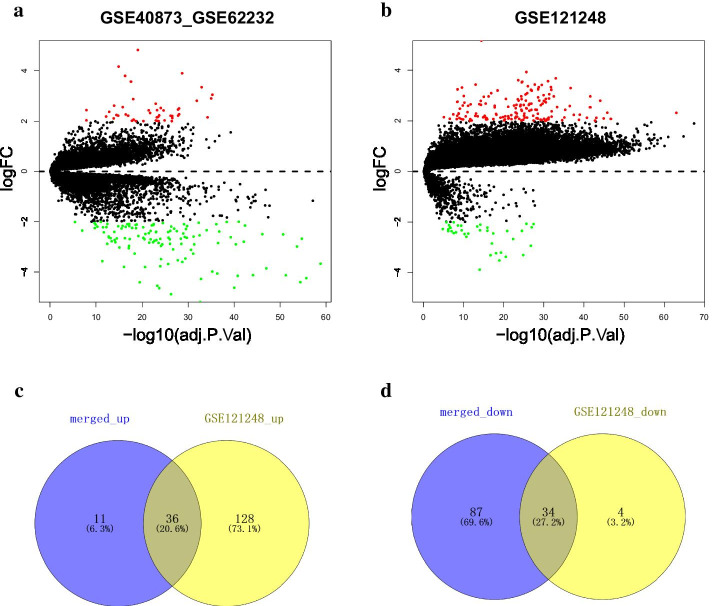
Differantially expressed genes. **a** Merged dataset generated from GSE62232 and GSE40873. **b** GSE121248. **c**,**d** Venn plot of shared gene between merged dataset and GSE121248

**Fig. 3:**
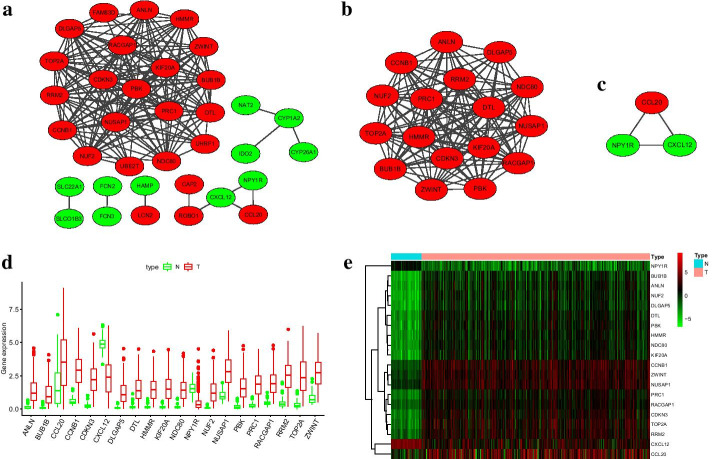
Hub genes. **a** PPI network was consisted of 35 genes, interaction score >  = 0.7 was the cutoff value. **b**,**c** Subnetwork 1 and subnetwork 2 identified by MCODE. Red represented upregulated gene and green represent downregulated gene. **d**,**e** Twenty hub genes were differencially expressed between tumor and nontumor samples in TCGA cohort (p < 0.001). “N” meant nontumor group and “T” meant tumor group

A total of 47 upregulated and 121 downegulated genes were identified from the merged dataset (Fig. 2a), and 164 upregulated and 38 downregulated genes were obtained from GSE121248 (Fig. 2b). 36 upregulated and 34 downregulated genes were further filtered through overlapping the DEGs of two datasets (Fig. 2c, d), which were used to construct PPI network. Thirty five genes were involved in the PPI network (Fig. 3a). Two subnetworks, which were regarded as hub genes, were further found and exhibited as 17 nodes and 135 edges in subnetwork 1 (Fig. 3b) and 3 nodes and 3 edges in subnetwork 2 (Fig. 3c).

### Expression of hub genes and functional enrichment in TCGA cohort

**Fig. 4:**
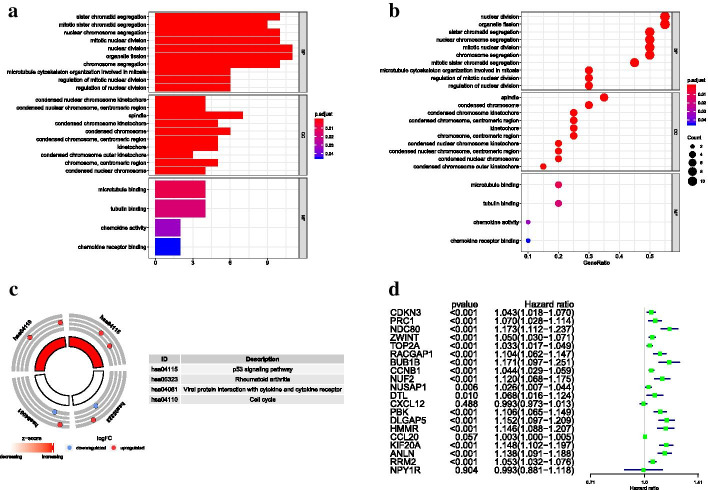
Functional enrichment and univariate regression analysis of hub genes in TCGA cohort. **a**,**b** GO enrichment analysis. CC, cellular component. BP, biological process. MF, molecular function. **c** Circle plot of KEGG pathway. **d** Univariate hazard regression analysis of hub genes

Expression of hub genes in TCGA cohort were analyzed, and the results exhibited that the level of all hub genes were significantly different between tumor and non-tumor samples (p < 0.001) (Fig. 3d, e). GO enrichment analysis showed that nuclear division and organelle fission were the most enriched GO terms (Fig. 4a, b), and hub genes were significantly enriched in p53 signaling pathway, Rheumatoid arthritis, Cell cycle and Viral protein interaction with cytokine and cytokine receptor pathways (Fig. 4c).

### Construction and predictive performance evaluation of the prognostic model

**Fig. 5:**
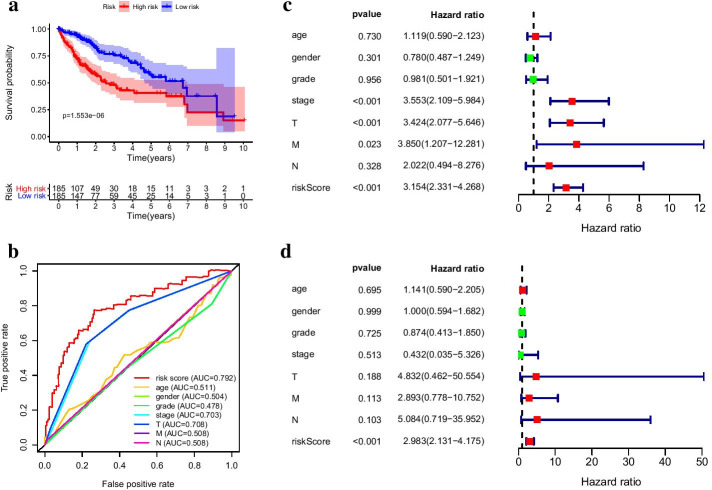
Predictive performance of prognostic model. **a** KM survival curve. **b** ROC curve of multiple indicators. **c** Univariate hazard regression analysis. **d** Multivariate hazard regression analysis

**Fig. 6:**
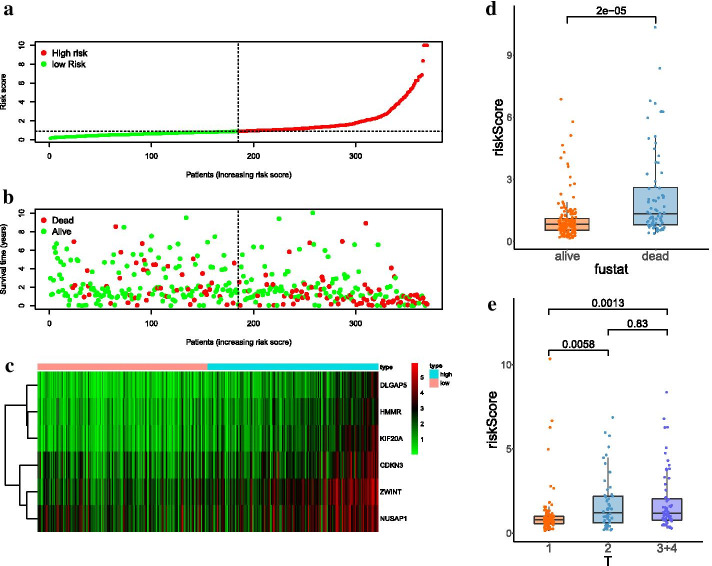
Risk score analysis. **a** Samples were sorted according to risk score from low to high. **b** Correlation between survical time and risk score. **c** Heatmap of six genes expression involved in prognosis model. **d** Correlation between risk score and fustate. **e** Box plot of risk score relative to tumor size

Seventeen hub genes in subnetwork 1 were applied to construct the prognostic model, while three genes in subnetwork 2 were discarded because they were not independent prognostic factors (p > 0.05) (Fig. 4d). Finally, the prognostic model involved six genes, and risk score = 0.65619 * *KIF20A*—0.40871 * *CDKN3* + 0.391238 * *ZWINT* − 1.07861 * *NUSAP1* + 0.757771 * *DLGAP5* + 0.479682 * *HMMR*. The detailed information was shown in Table 2. The risk scores of HCC patients were calculated according to the prognostic model, and the median of risk score was defined as the cutoff to divide patients into high- and low-risk groups (n = 370, which have complete survival state and risk score information). KM survival analysis showed low-risk group represented survival advantage compared with high-risk group (p = 1.553e−06) (Fig. 5a). ROC curve revealed that the AUC of risk score (AUC = 0.792) was higher than that of other clinical parameters (AUC = 0.511, 0.504, 0.478, 0.703, 0.708, 0.508, 0.508) (n = 235) (Fig. 5b). Univariate hazard regression analysis dispalyed that potential prognostic factors contained riskscore and several clinical indicators. However, only the satisfactory predictive performance of risk score persisted regardless of other clinical parameters in the multivariate hazard regression analysis (p < 0.001, n = 235, which have complete clinical and risk score information) (Fig. 5c, d). Risk score analysis illustrated that death cases were increased and survival time was incrementally reduced along with increased risk score (n = 370) (Fig. 6a–c). In adition, the risk score of death cases were significantly higher than that of alive individuals (p = 2.0e−05) (Fig. 6d), and the distribution of risk score relative to tumor size was displayed in Fig. 6e. These results suggested the potential significance of the prognostic model.

**Table 2:** Information of prognosis model

Gene_symble	Coef^#^	HR^#^	HR.95L	HR.95H	P value
CDKN3	− 0.40871	0.66451	0.47098	0.93754	0.01996
ZWINT	0.39124	1.47881	0.98122	2.22874	0.06157
NUSAP1	− 1.07861	0.34007	0.22714	0.50913	1.62e−07
DLGAP5	0.75777	2.13351	1.21285	3.75304	0.00855
HMMR	0.47968	1.61556	1.09716	2.37891	0.01512
KIF20A	0.65619	1.92743	1.23469	3.00886	0.00388

^#^ coef, coefficient; HR, hazard ratio

### Validation cohort

**Fig. 7:**
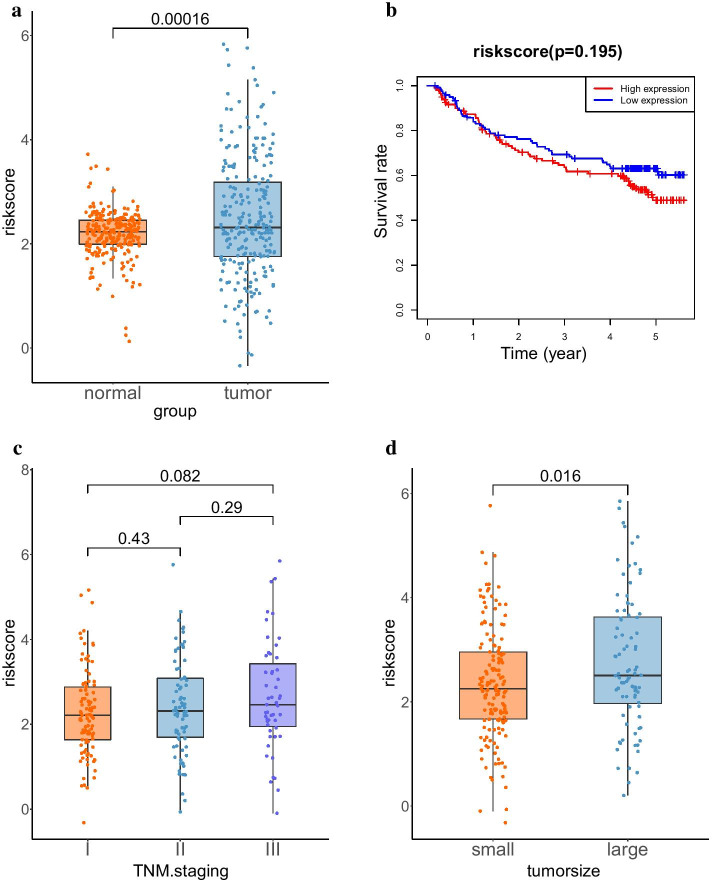
Results of validation cohort. **a** Comparison of risk score between tumor and normal samples. **b** KM survival curve, the cutoff divided tumor samples into two groups was the median of risk scores. **c** Distribution of risk score relative to tumor stage in HCC. **d** Comparison of risk score between small and large tumors. The diameter of 5 cm was the cutoff value

The predictive stability of the prognostic model was validated with GSE14520 dataset. The risk scores of tumor patients were significantly higher than that of the normal controls (Fig. 7a). KM survival analysis showed that the high-risk group displayed poorer survival compared with the low-risk group, while it did not reach statistical significance (Fig. 7b). Similarly, there was not significant correlation between risk score and TNM stage although the risk score gradually increased as the development of TNM stage (Fig. 7c). Tumor samples were divided into large and small groups with a diameter of 5 cm, and lower expression scores were significantly associated with smaller tumor size (Fig. 7d). These results suggested that the prognostic model may function as an independent biomarker to predict the outcome of patients with HCC.

## Discussion

Patients with HCC are generally characterized by poor prognosis, and there have been numerous studies to explore clinical biological signatures. In this study, three GEO datasets were used to analysis of DEGs. Subsequently, 35 genes were selected by PPI, and then twenty hub genes were generated by Cytoscape software. In order to explore the function of these hub genes, GO and KEGG enrichment analysis were carried out hosted on the TCGA cohort, and the results displayed that nuclear- and chromosome-related GO term, p53 signaling pathway and cell cycle were the main enrichment pathways. Seventeen genes (P < 0.05) were selected from twenty hub genes by univariate regression analysis to construct the prognostic model by COX hazard regression analysis using TCGA data, and finally, six genes (*CDKN3, ZWINT, NUSAP1, DLGAP5, HMMR, KIF20A*) were involved in the prognostic model.

*KIF20A* is associated with drug resistance and the clinical prognosis in diverse cancers. Previous studies suggest high expression of *KIF20A* is linked with poor clinical outcomes(14), and maybe involved in process of transformation of cirrhosis to HCC(15). In terms of drug resistance, *KIF20A* promotes paclitaxel resistance of breast cancer(16), and also insensitizes colorectal tumor to chemotherapy(17). In this study, the expression of *KIF20A* was positively correlated with the risk score that indicated poor outcomes.

*DLGAP5*, also known as *HURP*, is an important mediator for chromosome congression and alignment. Compelling evidence elucidates that *DLGAP5* promotes the development of non-small cell lung cancer(18), and is overexpressed in HCC and plays a critical role in the cancer cell cycle(19). Vice versa, a study confirms *DLGAP5* silence could inhibit HCC cell cycle and proliferation(20). We also suggested *DLGAP5* was a risk factor for HCC.

Many reports show that *ZWINT* is a predictor of tumor development. *ZWINT* is relative to risk index in pulmonary adenocarcinoma, that implies high level of *ZWINT* is correlated with poor outcomes(21). Similarly, elevated *ZWINT* could promote HCC clinicopathological features, and also possibly result in reduced overall survival and rising tumor recurrence(22). A study of prostate suggest *ZWINT* upregulation is correlated with higher Gleson scores and tumor grade(23).

The correlation between increased HMMR and poor prognosis has been reported in a variety of malignant tumors, including breast cancer(24), lung cancer(25), stomach cancer(26) and glioblastoma(27). Our results were in accordance with previous studies. In addition, *HMMR* may be contributed to proliferation, metastasis and invasion of breast cancer(28).

*CDKN3*, as tumor repressor, encodes protein that belongs to the dual-specificity protein phosphatase family. The role of *CDKN3* has been controversial in tumor progression. Increasing evidences suggest that *CDKN3* could promote tumor progression. Overexpression of *CDKN3* is associated with poor prognosis in lung adenocarcinoma(29), and the silence blocks proliferation and metastasis of pancreatic ductal adenocarcinoma(30). In contrast, *CDKN3* is relatively downregulated in brian tumor compared with normal brain tissue(31). In adition, the level of *CDKN3* is negatively correlated with HCC clinical pathological stage, and downregulation of *CDKN3* promotes tumor clonogenic ability(32). The present study was consistent with later that *CDKN3* was a protective factor in tumor development. The role of *CDKN3* in tumors needs to be further investigated.

The function of *NUSAP1*, the last signature of the prognostic model, has also been controversial in tumor progression. It’s reported that HCC patients with upregulated *NUSAP1* possess reduced survival times(33). Similar results are observed in a study of melanoma(34). Moreover, *NUSAP1* is involved in the resistance to antitumor therapy(35). However, current understanding of cervical cancer debates that low expression of *NUSAP1* is associated with higher tumor stage, and results in worse clinical outcomes(36). Our results illustrated the coefficient of *NUSAP1* was negative which implied the high level of *NUSAP1* predicted the survival advantage of HCC patients. The function of *NUSAP1* in tumor development need to be further explored by biomolecular and cellular research.

Finally, the predictive performance of the prognostic model was evaluated. K–M curve and risk score analysis indicated low-risk group had better prognosis than high-risk group, and univariate and multivariate regression analysis showed risk score might be an independent prognostic factor. Meanwhile, ROC analysis displayed the AUC of risk score was higher than that of other clinical indicators which illustrated risk score hold more prognostic value. In addition, the risk score of death cases were higher significantly than that of alive patients. The results of validation cohort also showed this prognostic model represented a prognostic significance for patients with HCC. Based on the analysis above, it was reasonable to regard risk score as a prognostic biomarker for HCC.

The whole process have combined multiple analysis methods, such as merging two GEO datasets and removing batch effect to expand the sample size and compensate for the lack of a certain sample type, generating subnetworks to identify hub genes rather than simply selecting top-ranked genes, and employing diverse risk score-related analysis and independent validation to evaluate the predictive power of the model. These methods may reduce false positive rate and therefore make the results more reliable and convincing. We hope that this workflow will be helpful for bioinformatics research in the future.

## Conclusion

We utilized bioinformatics methods to analyze HCC-related gene expression profiles from GEO and TCGA data. A prognostic model involving six genes was constructed through Cox hazard regression analysis, and the results of predictive performance evaluation represented the clinical value of this modle. At last, the consistent findings in validation cohort demonstrated that the prognostic model may be used as a tool to achive risk stratification of patients with HCC. For patients with higher risk score, more intensive systemic surveillance and therapy could be considered. Considering our attempt was definitely exploratory and the clinical value of the prognostic model to accurately predict prognosis was the ultimal goal, this work should not be regarded as the definitive result and more external verification work are needed to validate the predictive performance of this prognostic model.

## Data Availability

The datasets analyzed during this study are publicly available in GEO database at https://www.ncbi.nlm.nih.gov/geo/ and TCGA database at https://www.cancer.gov/about-nci/organization/ccg/research/structural-genomics/tcga.

## Abbreviations

DEG: Differentially expressed gene
FC: Fold Change
GEO: Gene expression omnibus
GO: Gene ontology
HCC: Hepatocellular carcinoma
KM: Kaplan–Meier
PPI: Protein–protein interaction
ROC: Receiver operating characteristic
TCGA: The Cancer Genome Atlas
